# Racial Health Equity and the Question of Black (Non?) Being: Exploring the Uses of Afropessimism in Approaches to Anti‐Racist Health Promotion

**DOI:** 10.1111/1468-4446.13232

**Published:** 2025-05-19

**Authors:** Tanisha Spratt

**Affiliations:** ^1^ Department of Global Health and Social Medicine King's College London London UK

## Abstract

Afropessimism is a critical framework that is often used to analyse anti‐Black violence and its deep entrenchment within systems and structures that perpetuate Black subjugation. By conceptualising Black life as ‘non‐life’, afropessimism examines how anti‐Black violence shapes health disparities, influencing who is deemed worthy of care and underscoring the systemic nature of this (d)evaluation. This framework holds significant potential for anti‐racist efforts that aim to address Black health disparities by exposing their root causes. However, afropessimism's central claim—that Black people are not only excluded from the category of the ‘human’ but are also positioned as its antithesis—poses challenges for anti‐racist strategies focussed on affirming recognitions of Black humanity to achieve health equity. This paper critically investigates the role of afropessimism in anti‐racist health promotion by examining divergent perspectives within its schools of thought. While all scholars who use afropessimist frameworks critically interrogate the systemic inequities that harm Black populations, they differ in their views on the potential for Black life within and beyond current anti‐Black systems and structures. These differences lead to varying implications for advancing anti‐racist health initiatives and promoting health justice through afropessimism. By analysing how afropessimism may inform anti‐racist health frameworks, this paper explores how its distinct theoretical perspectives can enrich, challenge, and constrain efforts to dismantle racial health inequities.

## 1 Introduction

Afropessimism is a concept that is widely used to examine contemporary practices of anti‐Black violence and how those practices are embedded within histories of anti‐Black subjugation. When used as anti‐racist praxis, afropessimism offers a way to conceptualise Black life as non‐life by considering the systemic permutation of anti‐Black violence in global contexts, and how that permutation increases Black peoples' vulnerability and susceptibility to poor health and premature death (Ajari 2023). In this way, afropessimism offers a potentially useful framework when seeking to articulate and conceptualise racial health disparities by pointing to how and why these disparities emerge.

However, as a theory that is predicated on the understanding that Black people are not only not human but that they also directly *antithesise* the human, afropessimism risks working against anti‐racist efforts that first call for a global recognition of Black humanity in their push to promote racial health equity. Following the murder of George Floyd in 2020, Black Lives Matter (BLM)—a global social justice organisation that calls for reform in anti‐Black policies and practice—gained notoriety for its call to promote Black life by pointing to its denial. By calling attention to the ways in which Black life is prematurely stunted through various forms of lethal discrimination, BLM advocates for a recognition of Black life as life to increase human protections that are typically afforded to other groups. In this way, afropessimism's denial of Black life as life—or the Black human as human—could be seen to work against the founding principles of anti‐racist movements that seek to promote Black life and minimise Black death. This article considers this juxtaposition by pointing to how afropessimism can be understood as a framework that both promotes racial health equity through its articulation of the harms generated by systemic anti‐Blackness and *negates* racial health equity through claims that the Black human is not human. In doing so, it asks the following questions: What are the uses of afropessimism in racial health justice work, and how do afropessimistic articulations of the Black non‐human engage with anti‐racist efforts to recognise racism as an embedded structure whilst allowing others to imagine alternative futures grounded in health equity and Black resistance?

These questions require further examination of *how* afropessimism is conceptualised, constructed, and applied and *by whom*. As with any social theory, the criteria used to determine who qualifies as an afropessimist are fluid, often leading to theorists being labelled as such even when they do not claim the label. There are canonical streams of afropessimism that point to Black non‐life as essential to modernity and offer bleak calculations on the likelihood of anti‐racist change (Warren 2018). There is also scholarship that aligns with key afropessimistic contentions but diverges from canonical applications of this framework by offering alternative strategies and solutions for change that are centred on resistance and imagining alternative futures (Sharpe 2016; Hartman 2021). The key difference between the former and the latter lies in their utility in promoting and sustaining anti‐racist efforts. If canonical afropessimism's primary contribution lies in promoting a recognition of Black life as non‐life whilst bleakly pointing to the unlikelihood of change, it falls short of offering much‐needed strategies and solutions that drive and sustain anti‐racist efforts. These strategies and solutions are essential when it comes to addressing the question of what it means for Black people to live liveable lives in the present and within contexts that routinely disallow it. On the other hand, if afropessimism is adapted and applied in ways that both attend to recognitions of Black life as non‐life *and* point towards strategies for resistance it can offer a valuable anti‐racist framework that promotes Black life by calling attention to its disavowal.

This article considers the uses of afropessimism in racial health equity work, with a particular focus on different streams of afropessimism that simultaneously highlight the pervasive nature of systemic inequities that harm Black people and attend to the question of Black (non‐) life. Focussing on scholarship predominantly located in the US and the UK whilst acknowledging its roots in decolonial thought from the Global South, I explore how afropessimism contains its own contradictions—calling for Black (non‐)life to be recognised as worthy of mourning while simultaneously arguing the Black life is positioned as antithetical to human life. In doing so, I critically attend to the discord between afropessimism's focus on highlighting racial health inequities and its insistence on configuring the Black non‐human, which necessitates understanding Black people outside of normative frameworks that seek to advance health equity.

Health inequities are upheld and perpetuated by systems and institutions that affirm afropessimism's ethos through Black negation. By introducing ideas of how the Black ‘non‐human’ is configured, and how we might live with and through this configuration in anti‐Black contexts, I argue in favour of theories that adopt aspects of afropessimism for its forward‐thinking possibilities. In doing so, I think alongside Black feminist scholars such as Christina Sharpe (2016), (2023), Saidiya Hartman (2021), and Jennifer Nash (2024) who, in different ways, draw on afropessimism whilst critiquing its limitations. I further highlight political orientations that centre Black joy and the cultivation of beauty as resistance and repair work, showing how they draw on afropessimistic logics to both juxtapose and foreground their work.

## 2 Configuring the Black ‘Non‐Human’

Sylvia Wynter's theorisation of the ‘genre of the human’ (Wynter 2003) has been instrumental in shaping contemporary afropessimist interpretations of the Black ‘non‐human’. In his book *Afropessimism* Frank Wilderson distinguishes between categories of the ‘human’ and the ‘non‐human’ (Wilderson 2020). The former represents people who are extended care, dignity, and inalienable rights and the latter refers to the people who are denied them. When elaborating further Wilderson notes that the ‘human’ is a category reserved for people who are not Black, and the ‘non‐human’ is a category assigned to people who are Black. The latter relegation does not deny Black peoples' material reality—Black people are, like all other people, real in an embodied sense of the term. Rather, it confers an understanding that Black life exists outside the boundaries of the human—antithesising the human—which disadvantages Black people in multiple facets of everyday life. If a person is not believed to be human they are unlikely to receive human considerations when it comes to healthcare, educational opportunities, workplace provisions, and the numerous other social determinants of health that govern and determine health and wellbeing (Butler 2021). According to Wilderson, Black people exist as ‘sentient beings against which humanity is defined’ (Wilderson 2020, 167) and are thus attended to in ways that often contravene human treatment. Yet, this human treatment is necessary to promote Black life and imagine Black futures in societies that routinely suppress Black life through draconian laws, policies, violent interventions, and interpersonal interactions.

The health consequences of anti‐Black racism are multifaceted. People who experience forms of racism‐induced chronic stress can experience persistent activation of the hypothalamic‐pituitary‐adrenal (HPA) axis, which initiates physiological responses to stress and increases their risk of developing numerous stress‐related health conditions including hypertension, anxiety, metabolic disorders, stroke, and depression (Aguilera 2011; Woods‐Burnham et al. 2020). Racism‐induced chronic stress can further generate ‘multi‐systemic physiological wear‐and‐tear through long‐term exposure to stress‐induced fluctuations or elevations in neuroendocrine response’ (Das 2013, 76). This process is commonly understood as ‘weathering’, a concept used to describe the cumulative health impacts of chronic exposure to racism (Geronimus 2023).

During the Covid‐19 pandemic, weathering was routinely used to explain racial and ethnic disparities in serious illness and death from the virus, with researchers attributing the heightened vulnerability of racially and ethnically minoritised groups to the cumulative health effects of pre‐exposure to systemic racism (Wakeel and Njoku 2021). According to Arline Geronimus and colleagues, ‘[b]ecause the stress response disrupts regulation of various systems throughout the body—for example, the cardiovascular, metabolic, and immune systems—the concept of weathering encompasses multiple systems and includes impacts on them that might not yet register clinically’ (Geronimus et al. 2006, 826). As such, racism‐induced stressors should be understood as key social determinants of health that can biologically accumulate *and* remain clinically undetected, indiscriminately manifesting through symptoms that may prohibit Black people from achieving ‘good health’.

As well as influencing Black health through experiential exposure, research shows that merely *anticipating* racism can increase one's risk of stress‐related health conditions. In their study on racial and ethnic disparities in hypertension prevalence, Margaret Hicken and colleagues found that racism‐related vigilance ‘is an important determinant of hypertension in Blacks (and perhaps Hispanics) through the continual activation of the biological stress response systems (e.g., autonomic and [HPA] systems) characteristic of this type of anticipatory and perseverative stress’ (Hicken et al. 2014, 122). Similarly, in their study on the health implications of stop and frisk policies in overpoliced Black communities, Naa Oyo Kwate and Shatema Threadcraft found that vigilance, anticipatory stress ‘and the repeated cognitive engagement of stressors is associated with a number of [health] outcomes including depressive symptoms, sleep difficulties, hypertension, and poor psychological health’ (Kwate and Threadcraft 2017, 545). These findings highlight the importance of measuring not only the biological effects of racism on health outcomes, but also its influence on the health‐promoting and health‐harming behaviours that *shape* those outcomes.

This dual focus on biology and behaviour resonates with afropessimism by engaging with both the systemic and existential dimensions of anti‐Blackness, illustrating how these interconnected forces shape health outcomes. Importantly, afropessimism highlights the role that health institutions, social structures, and systems of governance play in negating Black humanity and perpetuating health inequities and does not generate those inequities by revealing these practices. In his description of afropessimism as a framework to locate and understand contemporary configurations of Black (non) life Wilderson writes,

Black people are political currency or object, not political actors or subjects. Subjects have homes, or at least the capacity for some sort of sanctuary. Objects exist as implements, tools, in the psychic life of Human subjects … We are a species of sentient beings that cannot be injured or murdered, for that matter, because we are dead to the world

(Wilderson 2020, 198–9).

By describing Black people as ‘sentient beings’, Wilderson points to how Black people are thought of as *feeling* but not *living* in the ways that ‘non‐Black’ people are. This emphasis on feeling, in turn, signals a primitive recognition of Blackness that was constituted by and through the transatlantic slave trade, where slavery was routinely justified based on the presumption that hard labour and violent paternalism was ‘good’ for Black people because of their perceived ‘child‐like’ disposition and lack of agency (BBC 2014). During this time, Black people were not recognised as human in their capacity for intellect or reasoned judgement but could be understood as *feeling* because of their human responses to inhuman conditions. When enslaved people cried in response to brutal beatings or screamed during experimental surgical procedures that were performed on them without anaesthetic (Cronin 2020) they showed their capacity for feeling in ways that directly contradicted widespread beliefs that Black people either do not feel pain or feel pain to a lesser extent than white people (Ray 2023).

Decolonial and anti‐racist scholars routinely probe perceptions of Black animality when analysing ‘the human’ as a boundaried category. In *Becoming Human: Matter and Meaning in an Antiblack World* Zakiyyah Iman Jackson argues that rectifying the claim that Black people are animalistic by emphasising their humanity is ineffective when remedying anti‐Black racism because ‘[r]ecognition of personhood and humanity does not annul the animalisation of blackness’ (Jackson 2020, 17). In doing so, she points to the need to consider how we might benefit from ‘the rupture of “the human”’ when that categorisation fails to offer protections to Black people (Jackson 2020, 20). This ‘rupture’ both acknowledges Black humanity and generates new possibilities for imagining alternative futures for Black people who are human but do not benefit from that acknowledgement. Alexander Weheliye conceptualises this ‘rupture’ differently through his theory of ‘racializing assemblages’, which examines how race functions as a set of sociopolitical processes that delineate who is recognised as fully human, not‐quite‐human, or non‐human (Weheliye 2014). Unlike Jackson, Weheliye does not take as his starting point the objective truth of Black humanity but rather shows how humanity is constructed through racialised logics that expel certain groups from this classification. In doing so, he echoes Sylvia Wynter's earlier claim that colonisation produced a separation between the racialised ‘Human other’ and the ‘normal human Man’ (Wynter 2003, 265).

While antiblackness and afropessimism can be understood as distinct categories (Emejulu 2022) they are linked through their shared focus on contemporary inequities that emerge from histories of racist violence. In *Antiblackness* João Costa Vargas and Moon‐Kie Jung bridge this connection by pointing to ‘the singularity of Black people's dehumanisation, antihumanization’ (Jung and Vargas 2021, 9). Echoing afropessimistic claims of Black exclusion, both authors contend that Blackness marks the boundary of the human and, in doing so, point to the foundational role of antiblackness in structuring modern social and political life. The principal distinction between antiblackness and afropessimism may lie in their respective capacities to effect meaningful change for Black communities. Antiblackness scholars might ‘acknowledge antiblackness as a constitutive part of the development and reality of care in the colonial and postcolonial world’ (Hirsch 2020, 317) while calling for change to social and political orders that perpetuate racial inequities. In this way, scholars such as Christina Sharpe and Saidiya Hartman who offer forward‐thinking possibilities for Black people might be recognised as antiblackness scholars and *not* afropessimists. For canonical afropessimists, racial inequities cannot be resolved through existing systems because those systems are built on the notion of Black non‐humanity (Warren 2018).

When confronting the racial politics that shape how humanity is constructed, some decolonial and anti‐racist scholars reject the category ‘human’ as a form of praxis. In *Fugitive Feminism*, Akwugu Emejulu advocates for a recognition of Black *non*‐humanity to advance Black possibilities. Because ‘the human is a construction of whiteness’, Emejulu argues, to ‘enjoy liberty, equality and respect requires one's positioning in and submission to whiteness’, which renders it untenable (Emejulu 2022, 23). What is needed, she argues, is a way to think of oneself ‘outside the human’ by utilising ambivalence and liminality to ‘escape the conditions of [Black] captivity’ (Emejulu 2022, 51–52). While Wilderson presents a nihilistic view of Black non‐humanity as inherently tied to Black abjection, Emejulu frames Black non‐humanity as a liberatory space. Both positions make clear the ongoing pertinence of anti‐Black racism in the present but differ in their views on agency. Wilderson sees Black non‐humanity as an unchangeable condition, while Emejulu frames it as a space for resistance and radical possibility.

In *Lose Your Mother: A Journey Along the Atlantic Slave Route*, Saidiya Hartman maps the trajectory between enslavement and present‐day racial inequalities through her conceptualisation of what she refers to as the ‘afterlife of slavery’. This concept politically rejects efforts to separate past and present racial injustices, which often cite legal racial progress as evidence of systemic racism's decline or elimination (Bonilla‐Silva 2022). Through its confluence of past and present racial injustices, the ‘afterlife of slavery’ presents these injustices as interwoven systems of mutually implicative attitudes and modes of treatment that have systematically disallowed Black life. The primary consequences of the afterlife of slavery—limited access to health and education, incarceration, and impoverishment (Hartman 2021, 6)—include factors that medical sociologists commonly refer to as the social determinants of health. These determinants, used to explore the incidence and impact of a range of health conditions and diseases, cover a spectrum of socially imposed and governed health risks that lead to higher disease incidence and premature death among vulnerable populations.

Racism is a key social determinant of health that differentially exposes people of colour to illness and premature death in majority white countries (Ramsoondar et al. 2023). In the US where unequal access to health promoting resources (such as health care and good quality education) is often determined along racial lines (Ray 2023), what is needed is a reckoning with how histories of anti‐Black subjugation persist in the present. Hartman's thesis sits in conversation with other Black theorists who contextualise contemporary Black life in relation to its violent histories. In her theorisations on the mathematics of Black life, Katherine McKittrick notes that ‘historically present anti‐black violence is repaired by reproducing knowledge about the black subjects that renders them less than human’ (McKittrick 2015, 18). In making this claim, McKittrick argues for a recognition of how humanising Black life that has previously been denied can remedy contemporary forms of racial injustice that are rooted in the past. This humanisation can take place through archival work that seeks to unearth, reconfigure, and reimagine what Black life was when it was not deemed worthy of qualitative interrogation by contemporary researchers. McKittrick argues that engaging with archival materials, which expose the systemic devaluation and erasure of Black life through the conspicuous absence of Black voices, facilitates the posthumous humanisation of the Black (non) human—a praxis Hartman terms ‘critical fabulation’ (Hartman 2008).

By arguing that Black life and human life are antithetical, and that this antithesis is a central building block of modernity, afropessimists situate their beliefs about the impossibility of Black life in ways that directly mirror how Black life was originally conceived. Blackness came into being as a tool to differentiate between (white) life and (Black) non‐life and has since been used to perpetuate that perceived difference. By affirming this distinction, afropessimists argue for a recognition of ‘sentience’ as a perceived mode of Black being and fear of the Black body as a key driver of anti‐Black action (Wilderson 2020). When reckoning with this idea of Black sentience it is important to question whether it perpetuates the injustices it seeks to highlight. If Black people are understood as sentient beings, and this understanding is seen to characterise contemporary Black life, how can we meaningfully attend to, and contend with, the truth of Black humanity? Put differently, to what extent does highlighting beliefs about Black sentience without offering any recourse to redress them maintain and reiterate the problem of racial inequity? This tension is particularly urgent in questions concerning Black health, where the denial of Black humanity has long justified medical exploitation, neglect, and disparities in care.

## 3 Afropessimism and Black Health

Wilderson opens *Afropessimism* by describing his experience of a psychotic episode brought on by his realisation that, as a Black person, he is not considered worthy of care (Wilderson 2020). Described as a ‘breakthrough’, this moment signified a shift from Wilderson's broader understanding of anti‐Blackness as a theoretical framework that explains Black abjection to one that directly implicates him by impacting his health. This impact was felt by Wilderson not only through uncaring medical interactions, but also through his mindbody response to the conditions that brought about his psychotic episode. Wilderson writes,

I was moaning. Sobbing. The crisp disposable sheet that lined the gurney rasped as I shifted. I sat up when they [doctor and nurse] came into the room … The gurney rattled as I shook and cried. They didn’t approach. They didn’t call for help, not for themselves nor for me, a monstrous aphasic too black for care. That’s how I saw them see me … Cluster bombs spiked in my heart. I clutched my chest and cried out. Did they take a step back? Is it your heart? The doctor asked. I wanted to laugh. The funny thing about a mouth is that it needs to close as well as open if a word is to be made. Mine wouldn’t close; if it closed, I knew it wouldn’t open. The hinges of my jaws made moans or howls but not words

(Wilderson 2020, 7).

Wilderson's recollection of the momentary rift between the actions of his mind and body—the former recognising the need for communication, the latter unable to meet that need—demonstrates a reckoning with the Black body that is and the Black body that is *imagined*. Despite experiencing acute pain and severe mental illness, Wilderson remains cognisant of the ways in which he is being perceived by the healthcare workers observing him and considers acting accordingly. These actions would require him to *speak* rather than *laugh* and communicate the location of his pain rather than moan or howl in response to it. Conscious of being perceived as a ‘monstrous aphasic too black for care’, Wilderson recognises that his health needs will not—and cannot—be met by those tasked with treating him. This, Wilderson later infers, is because Black life is situated beyond the boundaries of care, which are inhabited by those deemed ‘human’ or ‘non‐Black’.

Wilderson's perspective is supported by the lived experiences of many Black people and evidenced by persistent racial disparities in healthcare access and treatment. In the US, Black people experience poorer health outcomes on average than their white peers in several areas including maternal mortality, cardiovascular disease, and type 2 diabetes (Leitch et al. 2021; Cleveland Clinic 2022; Rodríguez and Campbell 2017). Research shows that racial disparities in health are linked to a range of factors including relative access to health promoting resources (i.e. safe housing, nutritious food, and exercise opportunities), healthy living environments (i.e. those free from harmful toxins or pollutants), and good quality healthcare (Ray 2023). When it comes to clinical interactions, health provider bias can act as a key driver of differential treatment across multiple health domains including, but not limited to, pain management, speciality care, and mental health services (Sacks 2018). Additionally, mistrust in healthcare institutions among racially minoritised patients that are rooted in histories of medical violence can decrease uptake of primary care interventions that aim to reduce morbidity and premature death (Powell et al. 2019).

In their US study of the association between perceived provider discrimination, health care status, and health status in racially minoritised groups, Chioun Lee and colleagues found that, because minority groups perceive greater provider discrimination, they are more likely to delay seeking health advice than their white counterparts (Lee et al. 2009). This, in turn, means that racially minoritised patients are more likely to access care at critical stages than people who do not face this barrier. Additionally, in their US study of racial health disparities in rates of cervical cancer, Ariel Washington and Jill Randall found that white women are twice as likely to be screened for cervical cancer than Black women, and that high levels of perceived discrimination in healthcare settings can result in Black women adopting vigilant coping mechanisms when seeking care (Washington and Randall 2023). These coping mechanisms can, in turn, generate additional health burdens because of the positive correlation between race‐related vigilance and stress‐related health conditions, such as hypertension (Hicken et al. 2014).

Research shows that higher rates of health care avoidance amongst racially minoritised groups because of health provider bias also extends to mental health care. In their UK study of perceived barriers to accessing mental health services for racially minoritised groups, Anjum Memon and colleagues found that Black and other minority ethnic groups are less likely than white British people to contact their primary care physicians when experiencing mental health issues (Memon et al. 2016). This is, in large part, due to perceived insensitivity, cultural naivety, and discrimination from healthcare providers during clinical interactions. Racially minoritised patients are also less likely to be prescribed antidepressants or be referred to specialist mental health services than their white peers, despite Afro‐Caribbean men being significantly more likely than other ethnic groups to experience higher incidence of psychotic disorders (Memon et al. 2016). When Black people with mental ill health *are* referred to specialist services in the UK, they are 40% more likely to be referred through the criminal justice system than their white peers (Race Equality Foundation 2020). This disparity, in turn, means that Black people are more likely to encounter mental health services when in crisis than at the point of early intervention.

Health equity can be understood as the principle and practice of ensuring that everyone has a fair opportunity to attain ‘good health’ regardless of their social or economic circumstances (Marmot 2016). As such, it offers a way of addressing the underlying structural determinants that drive health disparities. Realising health equity in practice necessitates a critical engagement with the mechanisms through which inequities are produced and maintained. As a framework that outlines the undercurrents that drive and sustain racial health inequities, afropessimism can offer useful insights into the persistence of anti‐Blackness as a structuring logic within social and political systems. When used to foreground activist work that aims to bridge the divide between racially minoritised patients and their healthcare providers (such as initiatives to enhance recruitment amongst racially minoritised healthcare workers) afropessimism can offer a foundation for envisioning new systems and structures that promote racial health equity. Yet despite these possibilities, afropessimism's rationale for inadequate care—namely that, because Black people exist outside the category of the ‘human,’ they are not seen to warrant care—offers a fatalistic understanding of (anti‐) Black futures that can stunt progressive action. If the category of the ‘human,’ and the care that comes with being recognised as such, excludes Black people, how can we make use of afropessimism to meaningfully address the health disparities that stem from unequal access to human health needs? Put differently, if systemic change is impossible within existing frameworks, what role can afropessimism play in efforts to combat the current social determinants of health that routinely diminish Black life?

These questions give rise to additional questions about the meaning of Black life in the present. For Black people tasked with acknowledging afropessimistic claims about systemic anti‐Blackness whilst simultaneously trying to live and thrive in the present, how can/should they balance discordant feelings of despair and optimism? And what does it mean to reconcile these two feelings in ways that recognise the need for, and strive towards, Black health? When grappling with this tension it is necessary to consider whether hope and resilience in Black life *can* coexist with entrenched anti‐Blackness, or if such optimism inherently contradicts afropessimistic ways of thinking. The view that Black optimism negates the fundamental principles of afropessimism is not shared by all afropessimists. In his reflections on the ‘social life of social death’ Jared Sexton argues that Black optimism ‘is not the negation of the negation that is afro‐pessimism, just as black social life does not negate black social death by inhabiting it and vitalising it. A living death is as much a death as it is a living’ (Sexton 2016, 69). In making this statement Sexton offers a view of afropessimism that places it *in relation* to Black optimism rather than *in opposition* to it. In a similar way to how Black social life functions within the context of Black social death, Black optimism and afropessimism are, according to Sexton, both mutually significant and mutually implicative in the present moment.

Unlike canonical forms of afropessimism that leave little room for Black optimism, Sexton insists on understanding Black optimism as key to Black existence. In doing so, he is joined by scholars such as Tina Campt (2017) and Robin D. G. Kelley (2003) whose work emphasises the generative aspects of Black life through visual mediums and cultural production. For Black people contending with both despair and optimism, the latter can sustain negative feelings associated with the former. This can, in turn, promote Black health in instances where hope mitigates experiences of dejection. In global anti‐Black contexts, hope for a better future has historically been used to both strengthen and sustain Black communities in their fight for social justice and equality (Hill‐Jarrett 2023). Within these contexts, hope both emerges from these struggles and exists in relation to them when used to promote and ensure Black futures. In the aftermath of George Floyd's murder in 2020 came a renewed emphasis on Black joy as a way to resist the dehumanising and damaging effects of video images depicting anti‐Black violence that were circulated online. This reframing conceptualised Black joy as a powerful counter‐narrative to pervasive representations of Black suffering and made way for an understanding of the capacity and need for joy when fighting for social justice. In their book *The Black Joy Project: A Visual and Literary Love Letter to How We Thrive* Kleaver Cruz describes Black joy as a catalyst for healing when managing the effects of anti‐Black violence. Describing the relationship between Black joy and Black anguish Kleaver writes,

[Black joy] consistently runs alongside the suffering, pain, trials, and tribulations that we have endured throughout history and that we endure today. It’s a battery in our backs that recharges and powers us through the worst obstacles we face. And then, at the moment of overcoming an obstacle, Black joy is there to celebrate the feat

(Cruz 2023, 1–2).

Black joy offers a holistic view of Black life by demonstrating how it exists beyond the confines of Black suffering. As a form of resistance, Black joy emerges both through that suffering and in response to it. Moreover, as a culturally grounded practice, Black joy re‐claims Black life from the viewpoint of the oppressor and enriches it with an organic representation of Black life in its entirety, one conditioned (but not determined) by anti‐Black racism. As such, Black joy could be seen to oppose afropessimism by responding to anti‐Black violence through cultural affirmation, while simultaneously drawing on its logic to foster the conditions for its emergence. By framing Black joy as intertwined with anti‐Black violence, Kleaver affirms Sexton's insistence on recognising Black optimism in relation to Black negation. For Kleaver, Black joy parallels Black pain by ‘re‐charging the batteries’ of those who are routinely subjected to anti‐Black violence and who, in their fight to promote change, utilise joy for emotional restoration and sustained resistance. By offering fuel to people who become weary about the prospects of Black life in contexts that systematically deny it, Kleaver further shows how Black joy can function as a health promoting resource when used to foster resilience and fortify communal bonds.

Despite agreeing on the co‐existence of Black optimism/joy and Black negation, Kleaver and Sexton differ in their approach to Black possibilities. For Sexton, past, present, and future Black people were/are/will be subjected to the same harms if current systems and structures are not overhauled and replaced. For Kleaver, widescale revolution is not a necessary part of promoting Black health in the present or envisioning Black health in the future. Working within current systems and structures, Cruz offers possibilities for health by shifting the focus from anti‐Black suffering to Black thriving. This shift works against afropessimism by countering both its fatalistic perspective and its insistence on the Black ‘non‐human’. Cruz is joined in these efforts by Black‐centred organisations that not only critique anti‐Black dehumanisation but also mobilise that critique to deliver urgent health care access and targeted interventions to Black people. Organisations like Black Minds Matter UK, The Black Health Alliance (Canada), Harriet's Apothecary (US), and Criola (Brazil) work at the intersection of racial justice and health to provide care for Black people exposed to anti‐Black violence. In doing so, they insist on the possibility of Black life and Black futures within existing anti‐Black structures, advocating for a transformative model of care that affirms Black humanity and the potential for collective healing despite systemic oppression.

Afropessimism is routinely criticised for its lack of political impetus, with critics arguing that it fails to offer any solutions to the issues it highlights. It is important to note that, as with any social theory, afropessimism is constructed, articulated, and applied in different ways by different theorists. As this article has shown, Sexton and Wilderson—although contemporaries—do not conceptualise the limits of afropessimism in the same way. Nor do they necessarily agree on the implications of afropessimism for political praxis. While Sexton emphasises the structural permanence of anti‐Blackness and its deep entanglement within social formations, Wilderson underscores the ontological irreconcilability between Black existence and humanity as it is currently configured. The former arguably leaves room for political resistance while the latter renders that political resistance futile in existing structures, necessitating complete systemic rupture.

In her review of *Afropessimism*, Gloria Wekker defies what she refers to as Wilderson's ‘lack of thinking in political terms and lack of willingness, or even inclination, to take political action’ (Wekker 2021, 93). This critique is grounded in Wekker's interpretation of the text as lacking in intersectional engagement and sufficient consideration of the wider Black diaspora (Wekker 2021, 94). While much of the foundational work on afropessimism has emerged from and in relation to the US, it is indebted to anti‐Blackness scholarship from theorists in the Global South. These theorists include Franz Fanon (2008), Ochy Curiel et al. (2016), and Achille Mbembe (2019), whose decolonial praxis shaped contemporary considerations of, and moves towards, afropessimism.

Wekker's critique is further grounded in Wilderson's assertion that his work aligns with other notable authors whose work focuses on Black social death. Critiquing what she views as Wilderson's misapplication of the works of scholars such as Saidiya Hartman, Wekker contends that *Afropessimism* embodies a ‘far‐reaching generalisation’ of those works and superficially adapts ideas that Wilderson selectively borrows from various authors (Wekker 2021, 94). This adaptation, Wekker argues, allows Wilderson to give these authors' ideas a ‘loveless and hopeless twist, which often goes against the intentions of the author in question’ (Wekker 2021, 94). In pointing to this ‘superficial adaptation’ of adjacent work, Wekker conveys the importance of dismantling the notion that afropessimism is a monolithic framework adopted by people who share the same ideas. Different so‐called afropessimists conceptualise the relationship between Black life and Black death in different ways. Recognising this is crucial when seeking to understand the limits and potentials of afropessimism in promoting Black health through envisioning anti‐racist futures.

## 4 Envisioning Anti‐Racist Futures Through Strategies for Health and Wellbeing

In her book *Viral Justice: How We Grow The World We Want* Ruha Benjamin emphasises the importance of envisioning alternative systems, structures, and spaces when engaging in various modes of resistance (Benjamin 2022). Benjamin's thesis offers a life‐affirming lens that critiques the world *as it is* to prompt consideration of the world *as it should be*. This solution‐based orientation—referred to by Benjamin as ‘world‐building’—uses afropessimist claims about the intrinsic nature of anti‐Black racism to current systems and structures as a vehicle for change‐making. It further offers a mode of thinking that centres joy and justice in its political ethos. By spotlighting the work of local activists who are currently redressing unequal systems within their communities through tailored initiatives, Benjamin shows how the change we want to see can be enacted by local change‐makers. As praxis, this work can form a type of ‘contagion’ (Fay july 2023) that gradually moves us towards the world we envision.

When presenting her thesis on world‐building, Benjamin emphasises the essential role of beauty in shaping visions of justice and equity, echoing Hartman's earlier assertion that ‘beauty is not a luxury’ (Hartman 2021). She argues that, like food, beauty is something we ‘hunger’ for, ‘which is why art, aesthetics, and imagination are vital to world‐building’ (Benjamin 2022, 55). In the context of racial health equity, cultivating beauty can act as a survival strategy in racist environments. By promoting creativity, joy, and cultural affirmation, it can empower marginalised communities to resist oppressive structures through self‐actualisation. Additionally, it can counter the nihilism rooted in canonical afropessimism by fostering care work through creative expression. In her book *Ordinary Notes* Christina Sharpe describes how her mother cultivated beauty at home to make living in an anti‐Black world possible. This cultivation proffered a recognition of Blackness that was orientated around care and affective pleasure rather than suffering, survival, or resilience, which often dominate narratives about Black life. Sharpe writes,

Knowing that every day that I left the house, many of the people whom I encountered did not think me precious and showed me so, my mother gave me space to be precious—as in vulnerable, as in cherished. It is through her that I first learned that beauty is a practice, that beauty is a method, and that a vessel is also ‘a person into whom some quality (such as grace) is infused’

(Sharpe 2023, 80).

By providing Sharpe with space to be precious and vulnerable her mother instilled in her an understanding of the need for both when surrounded by anti‐Black thought, rhetoric, and violence. Without this space, Sharpe suggests, she may not have been equipped to withstand the everyday violence she encountered from those who did not see her as precious or worthy of care. In this way, the beauty that Sharpe's mother enacted in this space offered Sharpe armament against the negative impacts of those encounters. By giving Sharpe ‘space to dream’, her mother provided her with the tools to envision alternative realities that directly countered her lived experience. At its core, beauty as anti‐racist method is a humanising project that highlights the uses of beauty in fostering care and resilience amongst marginalised groups (Hartman 2021). It is a survival strategy for those burdened by the harms of everyday racism and it can foster health through creative expression and cultural affirmation when articulated through crafting (Barber february 2025).

Through her work on what it means to live ‘in the wake’ of slavery Sharpe examines the relationship between Black social life and Black social death, building on Sexton's claim that they operate simultaneously while exploring ways to reconcile their apparent opposition. ‘In the midst of so much death and the fact of Black life as proximate to death’, Sharpe writes, ‘how do we attend to physical, social, and figurative death and also to the largeness that is Black life, Black life insisted from death?’ (2016, 17). This question is echoed in Fred Moten's earlier prompt to ‘consider exhaustion as a mode or form or way of life’ (Moten 2013) to better understand current possibilities for living. As a method for survival, beauty offers a way to counter anti‐Black dehumanisation by showing how Black life is being lived *despite* the oppressive forces that govern it.

As people living ‘in the wake’ of slavery and colonial violence, Black people resist anti‐Black violence through creating spaces and ideas that prioritise their health and wellbeing. While these spaces *emerge* from vulnerability, they are not *determined* by it. By transforming vulnerability into a site of generative potential, these spaces directly challenge the negation that afropessimism recognises as an immutable reality that stunts Black life. They also allow for relationality between Black people who recognise each other as more than the racism that governs their lives. As Sharpe writes, to be in the wake ‘is also to recognise the ways that we are constituted through and by continued vulnerability to overwhelming force though not *only* known to ourselves and to each other by that force’ (2016, 16).

## 5 Conclusion

Afropessimism's foundational claim that Black people are positioned as ‘non‐human’ presents a conceptual challenge to anti‐racist health equity initiatives grounded in affirmations of Black humanity within global frameworks. By highlighting the structures underpinning contemporary anti‐Blackness afropessimism offers an important framework through which to understand health inequities. However, through its inherent nihilism, afropessimism risks stunting ant‐racist political action. Theorists such as Wilderson illuminate the need for change but do not provide actionable strategies for promoting Black health in the present or achieving it in the future. Conversely, scholars who draw on afropessimism to theoretically ground their work highlight the uses of afropessimism in pointing to those changes. Sharpe, Hartman, and Benjamin utilise afropessimism for its forward‐thinking possibilities and, in doing so, highlight its uses in contemporary anti‐racist efforts. Through their work they further contribute towards those changes by creating strategies for resistance. These strategies foreground the need to find ways of living in the violent present whilst working towards non‐violent futures.

By crafting actionable strategies for resistance, these theorists allow us to recognise how change is currently being enacted *in response to* and *alongside* anti‐Black violence. There is a need for ongoing engagement with afropessimism to both leverage its critique of systemic violence and navigate the challenges it poses for constructing actionable pathways towards health equity and social justice. As noted by philosopher Orlando Hawkins, ‘Afropessimism's turn to nihilism, though illuminating of anti‐Blackness, must be balanced with a life‐affirming philosophy and revolutionary commitment that equips one to avoid resignation, despair, or suicide’ (Hawkins 2023, 2). Further research is needed to understand how afropessimism's critiques of systemic violence can be harnessed in the service of strategies that centre healing, resilience, and collective care. By engaging afropessimism not only as critique but also as a catalyst for creative resistance, scholars and activists can uncover new possibilities for advancing Black health, dignity, and social justice.

## Ethics Statement

The author has nothing to report.

## Consent

The author has nothing to report.

## Conflicts of Interest

The author declares no conflicts of interest.

## Permission to Reproduce Material From Other Sources

The author has nothing to report.

## Data Availability

The data that support the findings of this study are available in the supplementary material of this article.
